# Spectral derivation of the classic laws of wall-bounded turbulent flows

**DOI:** 10.1098/rspa.2017.0354

**Published:** 2017-08-09

**Authors:** Gustavo Gioia, Pinaki Chakraborty

**Affiliations:** Okinawa Institute of Science and Technology Graduate University, Onna-son, Okinawa 904-0495, Japan

**Keywords:** mean-velocity profile, turbulent energy spectrum, spectral link, wall turbulence, log law

## Abstract

We show that the classic laws of the mean-velocity profiles (MVPs) of wall-bounded turbulent flows—the ‘law of the wall,’ the ‘defect law’ and the ‘log law’—can be predicated on a sufficient condition with no manifest ties to the MVPs, namely that viscosity and finite turbulent domains have a depressive effect on the spectrum of turbulent energy. We also show that this sufficient condition is consistent with empirical data on the spectrum and may be deemed a general property of the energetics of wall turbulence. Our findings shed new light on the physical origin of the classic laws and their immediate offshoot, Prandtl’s theory of turbulent friction.

## 1. Introduction

The mean-velocity profile (MVP) of a wall-bounded turbulent flow is the function *u*(*y*) that gives the mean (time-averaged) velocity of the flow, *u*, at any given distance to the wall, *y* [1–3]. MVPs are used to compute fluxes and other quantities of engineering interest, notably the turbulent friction (which sets the power needed to operate a pipeline and the draining capacity of a waterway, for example); on that account, the MVPs of common wall-bounded turbulent flows, like pipe flow and boundary-layer flow, have long been the subject of research. The most influential research was carried out by L. Prandtl, who in the early twentieth century formulated the classic laws of the MVPs [1–3]. Known as the law of the wall, the defect law and the log law, the classic laws underly the customary division of MVPs into layers (the inner layer, the outer layer, the overlap layer), which in turn informs the very way in which MVPs are apprehended and conceptualized as macroscopic turbulent phenomena. Indeed, the classic laws have become so well established that they are scarcely put to the empirical test any longer; rather they are widely used to plot, collate and assess experimental, computational and atmospheric data on wall-bounded turbulent flows [2,3]. What is more, Prandtl’s theory of turbulent friction [1–3], a mainstay of hydraulic engineering, hydrology, meteorology and kindred disciplines, is but a corollary of the classic laws.

In the original work of Prandtl, the classic laws are essentially postulated as a suitable set of assumptions regarding the asymptotic behaviour of *u*(*y*) in the limits of vanishing viscosity and infinite turbulent domain. Nevertheless, the classic laws have been shown to be consistent with models of wall-bounded turbulent flows, notably the attached-eddy hypothesis [4,5]. Here we seek to relate the classic laws to the turbulent eddies (or fluctuations) of a flow, the carriers of the flow’s turbulent energy, without having recourse to any specific model of those eddies. To that end, we shall rely on the ‘spectral analogues’ [6] (or, more properly, the ‘spectral analogues of the classic laws,’ which are similar to the classic laws but apply to the spectrum of turbulent energy instead of the MVPs) and the ‘spectral link’ [7] (which furnishes a connection between the MVPs and the spectrum of turbulent energy). The spectrum of turbulent energy is a function of the wavenumber *k*, *E*(*k*), the physical significance of which can be grasped from the expression $v_{s}^{2}=\int_{1/s}^{\infty }E(k)\;dk$ [2], where *v*_*s*_ is the characteristic velocity, and $v_{s}^{2}$ the kinetic energy per unit mass, associated with an eddy of size *s*. Thus *E*(*k*), which may be readily measured experimentally, represents the way in which turbulent energy is allotted among eddies of different sizes in a flow.

As it turns out, a derivation of the classic laws can be completed, starting from the spectral analogues and the spectral link, if *E*(*k*) satisfies a sufficient condition that spells out the general scope of viscous and finite-domain effects in the energetics of wall turbulence. Our task will be to identify, and put to the empirical test, that sufficient condition.

## 2. The classic laws

We begin by going over the standard derivation of the classic laws [2,3], which starts with a dimensional analysis. The dimensional variables are 6: *y*, *u*′≡*du*/*dy* (note that *u* is not Galilean invariant, thus the choice of *u*′), *ρ*, *ν*, *τ*_w_ and *δ*. Here, *ρ* is the density of the fluid, *ν* is the kinematic viscosity of the fluid, *τ*_w_ is the shear stress at the wall (i.e. the shear force that the flow exerts on the wall, per unit area of wall), and *δ* is the size of the turbulent domain (in pipe flow, for example, *δ* is customarily taken to be the radius of the pipe; more generally, *δ* is chosen so that the mean velocity peaks at *y*=*δ*). From Buckingham’s *Π*-theorem [8] and the dimensional equations [*u*′]=[*y*]^−1^[*τ*_w_]^1/2^[*ρ*]^−1/2^, [*δ*]=[*y*][*τ*_w_]^0^[*ρ*]^0^ and [*ν*]=[*y*]^1^[*τ*_w_]^1/2^[*ρ*]^−1/2^, we conclude that the functional relation among the six dimensional variables can be expressed as an equivalent functional relation among three dimensionless variables: *yu*′/*u*_*τ*_, *y*/*δ* and *yu*_*τ*_/*ν*, where *u*_*τ*_ is the frictional velocity, *u*_*τ*_≡(*τ*_w_/*ρ*)^1/2^. It follows that *yu*′/*u*_*τ*_=*F*(*y*/*δ*,*yu*_*τ*_/*ν*), which can also be written as

2.1 $$\tilde{y}{\tilde{u}}^{\prime }=F(\hat{y},\tilde{y}),$$

where *F* is an unknown function, $\tilde{u}\equiv u/u_{\tau }$, ${\tilde{u}}^{\prime }\equiv \partial \tilde{u}/\partial \tilde{y}$,

2.2 $$\hat{y}\equiv \frac{y}{\delta }\;\mathrm{and}\;\tilde{y}\equiv \frac{yu_{\tau }}{\nu }.$$

If we assume that *F* becomes independent of $\hat{y}$ for $\hat{y}\to 0$ (the limit of infinite domain), we obtain the law of the wall:

2.3 $$\lim_{\hat{y}\to 0}\tilde{y}{\tilde{u}}^{\prime }=F_{w}(\tilde{y}),$$

where $F_{w}(\tilde{y})\equiv \lim_{\hat{y}\to 0}F(\hat{y},\tilde{y})$. If we assume that *F* becomes independent of $\tilde{y}$ for $\tilde{y}\to \infty$ (the limit of vanishing viscosity), we obtain the defect law:

2.4 $$\lim_{\tilde{y}\to \infty }\tilde{y}{\tilde{u}}^{\prime }=F_{d}(\hat{y}),$$

where $F_{d}(\hat{y})\equiv \lim_{\tilde{y}\to \infty }F(\hat{y},\tilde{y})$. If we assume that *F* becomes independent of both $\hat{y}$ and $\tilde{y}$ for $\hat{y}\to 0$ and $\tilde{y}\to \infty$ (the limit of infinite domain and vanishing viscosity), we obtain the log law:

2.5 $$\lim_{\hat{y}\to 0\;\tilde{y}\to \infty }\tilde{y}{\tilde{u}}^{\prime }=\kappa^{-1},$$

where $\kappa^{-1}\equiv \lim_{\hat{y}\to 0\;\tilde{y}\to \infty }F(\hat{y},\tilde{y})$ is a dimensionless constant (the inverse of the Kármán constant, *κ*).

## 3. The spectral analogues

To derive the spectral analogues, we follow the same steps as in the standard derivation of the classic laws. The dimensional variables are *E*, *k*, *y*, *δ*, *τ*_w_, *ρ* and *ν*. From Buckingham’s *Π*-theorem and the dimensional equations [*E*]=[*y*]^1^[*τ*_w_]^1^[*ρ*]^−1^, [*k*]=[*y*]^−1^[*τ*_w_]^0^[*ρ*]^0^, [*δ*]=[*y*]^1^[*τ*_w_]^0^[*ρ*]^0^ and [*ν*]=[*y*]^1^[*τ*_w_]^1/2^[*ρ*]^−1/2^, we conclude that the functional relation among the seven dimensional variables can be expressed as an equivalent functional relation among four dimensionless variables ($E/yu_{\tau }^{2}$, *ky*, *y*/*δ* and *yu*_*τ*_/*ν*), in the form

3.1 $$\frac{E}{yu_{\tau }^{2}}=f(ky,\hat{y},\tilde{y}).$$

If we assume that *f* becomes independent of $\hat{y}$ in the limit of infinite domain, we obtain the spectral analogue of the law of the wall:

3.2 $$\lim_{\hat{y}\to 0}\frac{E}{yu_{\tau }^{2}}=f_{w}(ky,\tilde{y})\equiv \lim_{\hat{y}\to 0}f(ky,\hat{y},\tilde{y}).$$

If we assume that *f* becomes independent of $\tilde{y}$ in the limit of vanishing viscosity, we obtain the spectral analogue of the defect law:

3.3 $$\lim_{\tilde{y}\to \infty }\frac{E}{yu_{\tau }^{2}}=f_{d}(ky,\hat{y})\equiv \lim_{\tilde{y}\to \infty }f(ky,\hat{y},\tilde{y}).$$

If we assume that *f* becomes independent of both $\hat{y}$ and $\tilde{y}$ in the limit of infinite domain and vanishing viscosity, we obtain the spectral analogue of the log law:

3.4 $$\lim_{\hat{y}\to 0\;\tilde{y}\to \infty }\frac{E}{yu_{\tau }^{2}}=f_{l}(ky)\equiv \lim_{\hat{y}\to 0\;\tilde{y}\to \infty }f(ky,\hat{y},\tilde{y}).$$

For future reference, it bears emphasis that the spectral analogues entail mere pointwise convergence [9] in (3.2)–(3.4), just as the classic laws entail mere pointwise convergence in (2.3)–(2.5). Thus, for example, the spectral analogue of the law of the wall can be stated as ‘$f(ky,\hat{y},\tilde{y})$ converges pointwise to $f_{w}(ky,\tilde{y})$ for $\hat{y}\to 0$.’

## 4. The spectral link

**Figure 1.**
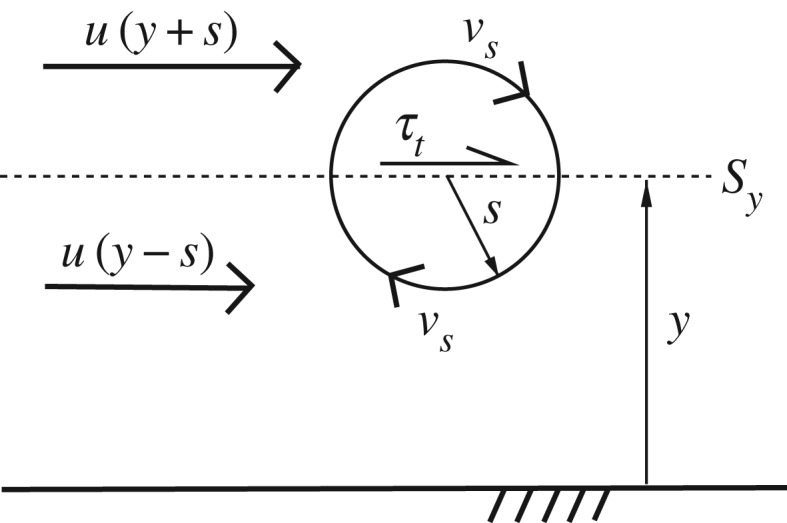
Schematic for the derivation of the spectral-link formula for the turbulent shear stress *τ*_t_ (after [7]). A turbulent eddy of size *s* and velocity *v*_*s*_ straddles wetted surface *S*_*y*_ at a distance *y* from the wall. The eddy picks up high-momentum fluid from above *S*_*y*_ and advects it downwards through *S*_*y*_ at a rate ∝*v*_*s*_. At the same time, the eddy picks up low-momentum fluid from below *S*_*y*_ and advects it upwards through *S*_*y*_ at a rate ∝*v*_*s*_. The net flux of momentum through *S*_*y*_ equals the turbulent shear stress that the eddy produces on *S*_*y*_; thus, *τ*_t_∝*ρu*′*sv*_*s*_ (where we have substituted *u*′*s* for *u*(*y*+*s*)−*u*(*y*−*s*)). Since *v*_*s*_ is an increasing function of *s* (because $v_{s}^{2}=\int_{1/s}^{\infty }E(k)\;dk$ and *E*(*k*)>0 for all *k*), the production of *τ*_t_ on *S*_*y*_ is dominated by eddies of size *y* and velocity *v*_*y*_—that is, by the largest eddies that straddle *S*_*y*_—and *τ*_t_=*cρu*′*yv*_*y*_, where *c* is a dimensionless proportionality constant.

We now turn to the spectral link. Crucial to the spectral link is a formula, the derivation of which we relegate to the caption of figure 1, that expresses the turbulent shear stress at a distance *y* from the wall, *τ*_t_, in terms of the velocity of an eddy of size *y*, *v*_*y*_, in the form *τ*_t_=*cρu*′*yv*_*y*_, where *c* is a dimensionless constant. That formula for *τ*_t_ may be combined with the equation of momentum balance, *τ*_t_+*ρνu*′=*τ*_w_(1−*y*/*δ*) [2,3] (where *ρνu*′ is the viscous shear stress and *τ*_w_(1−*y*/*δ*) is the total shear stress), to obtain an expression for *u*′ that links the MVPs to the spectrum of turbulent energy:

4.1 $$\tilde{y}{\tilde{u}}^{\prime }=\frac{1-\hat{y}}{{\tilde{y}}^{-1}+c{\tilde{v}}_{y}},$$

where

4.2 $${\tilde{v}}_{y}^{2}\equiv {\left(\frac{v_{y}}{u_{\tau }}\right)}^{2}=u_{\tau }^{-2}\int_{1/y}^{\infty }E(k)\;dk.$$

## 5. Analysis

In principle, (4.1)–(4.2) can be used to compute MVPs for any specific model of the spectrum. For the phenomenological model of Kolmogorov [2,3,10,11], in which a power-law spectrum that is independent of both *δ* and *ν* is multiplied by a conventional correction for the effect of finite domain and by a conventional correction for the effect of viscosity, it has been shown that (4.1)–(4.2) yield MVPs complete with all of the distinctive features known from empirical data on wall-bounded turbulent flows, including buffer layers (which turn out to stem from the multiplicative correction for the effect of viscosity) and wakes (which turn out to stem from the multiplicative correction for the effect of finite domain) [7,12]. What is more, the MVPs attendant on the phenomenological model satisfy the classic laws [7]. And yet, as a specific model of the spectrum, the phenomenological model can hardly serve as the foundation of broadly applicable laws. Thus, in what follows, rather than focusing on any specific model, we shall seek to ascertain a minimally restrictive set of constraints on the spectrum under which (4.1)–(4.2) can be guaranteed to yield MVPs that satisfy the classic laws.

We start by substituting (3.1) into (4.2) and changing the integration variable to *ξ*≡*ky*:

5.1 $${\tilde{v}}_{y}^{2}=\int_{1}^{\infty }f(\xi ,\hat{y},\tilde{y})\;d\xi .$$

Consider, for example, the limit of infinite domain, $\hat{y}\to 0$, and suppose that we can write

5.2 $$\lim_{\hat{y}\to 0}{\tilde{v}}_{y}^{2}=\lim_{\hat{y}\to 0}\int_{1}^{\infty }f(\xi ,\hat{y},\tilde{y})\;d\xi =\int_{1}^{\infty }\lim_{\hat{y}\to 0}f(\xi ,\hat{y},\tilde{y})\;d\xi .$$

In this case, we would be able to invoke the spectral analogue of the law of the wall (whereby $f(\xi ,\hat{y},\tilde{y})$ converges *pointwise* to $f_{w}(\xi ,\tilde{y})$ for $\hat{y}\to 0$) and conclude that ${\tilde{v}}_{y}^{2}$ becomes independent of $\hat{y}$ in the limit of infinite domain and, consequently, that (4.1) reduces to (2.3), the law of the wall, in that limit. Note, however, that it might not be possible to bring the limit inside the integral, as we have done in (5.2), unless $f(\xi ,\hat{y},\tilde{y})$ converges *uniformly* to $f_{w}(\xi ,\tilde{y})$ for $\hat{y}\to 0$ [9]. This brings us to the nub of the argument:

Let us assume that *the effect of finite domain and the effect of viscosity is to depress the dimensionless spectrum*, at least for *ky*≥1 (the domain of integration in (5.1)):

5.3 $$\begin{array}{rl} 0\leq f(ky,\hat{y},\tilde{y}) & \leq f_{w}(ky,\tilde{y})\;\leq \;f_{l}(ky)\\ \;\text{and}\;\;0\leq f(ky,\hat{y},\tilde{y}) & \leq f_{d}(ky,\hat{y})\;\leq \;f_{l}(ky), \end{array}\}$$

for *ky*≥1. Then, convergence in (3.2)–(3.4) is uniform and, consequently, there exist functions $F^{2}(\hat{y},\tilde{y})$, $F_{w}^{2}(\tilde{y})$ and $F_{d}^{2}(\hat{y})$ such that

5.4 $$\begin{array}{rl} F^{2}(\hat{y},\tilde{y}) & \equiv \int_{1}^{\infty }f(\xi ,\hat{y},\tilde{y})\;d\xi , \end{array}$$

5.5 $$\begin{array}{rl} \lim_{\hat{y}\to 0}F^{2}(\hat{y},\tilde{y})=F_{w}^{2}(\tilde{y}) & \equiv \int_{1}^{\infty }f_{w}(\xi ,\tilde{y})\;d\xi , \end{array}$$

5.6 $$\begin{array}{rl} \lim_{\tilde{y}\to \infty }F^{2}(\hat{y},\tilde{y})=F_{d}^{2}(\hat{y}) & \equiv \int_{1}^{\infty }f_{d}(\xi ,\hat{y})\;d\xi \end{array}$$

5.7 $$\begin{array}{rl} \;\text{and}\;\lim_{\hat{y}\to 0\;\tilde{y}\to \infty }F^{2}(\hat{y},\tilde{y}) & =C^{2}, \end{array}$$

where $C^{2}\equiv \int_{1}^{\infty }f_{l}(\xi )\;d\xi$ [9]. In this case, we can combine (5.4) with (5.1) to write ${\tilde{v}}_{y}=F(\hat{y},\tilde{y})$, which we substitute in (4.1), with the result:

5.8 $$\tilde{y}{\tilde{u}}^{\prime }=\frac{1-\hat{y}}{{\tilde{y}}^{-1}+cF(\hat{y},\tilde{y})}.$$

This latter equation should be compared with (2.1), the equation from which the classic laws are customarily derived, as we have seen, by making ad hoc assumptions on the asymptotes of $F(\hat{y},\tilde{y})$, a function on which nothing is known apart from what might be inferred from (2.1). By contrast, function $F(\hat{y},\tilde{y})$ can be computed as an integral of the spectrum, using (5.4), and carries a physical meaning independent of (5.8). Indeed, $F(\hat{y},\tilde{y})$ equals ${\tilde{v}}_{y}$, the dimensionless velocity of the eddies that dominate the production of turbulent shear stress at a distance *y* from the wall (cf. the caption of figure 1). What is more, provided that the spectrum satisfies condition (5.3), the asymptotes of $F(\hat{y},\tilde{y})$ are guaranteed to be those of (5.5)–(5.7). Thus, if the spectrum satisfies condition (5.3), we can invoke (5.5) and (5.8) to conclude that

5.9 $$\lim_{\hat{y}\to 0}\tilde{y}{\tilde{u}}^{\prime }=\frac{1}{{\tilde{y}}^{-1}+cF_{w}(\tilde{y})},$$

which we recognize as the law of the wall (cf. equation (2.3)). If the spectrum satisfies condition (5.3), we can invoke (5.6) and (5.8) to conclude that

5.10 $$\lim_{\tilde{y}\to \infty }\tilde{y}{\tilde{u}}^{\prime }=\frac{1-\hat{y}}{cF_{d}(\hat{y})},$$

which we recognize as the defect law (cf. equation (2.4)). If the spectrum satisfies condition (5.3), we can invoke (5.7) and (5.8) to conclude that

5.11 $$\lim_{\hat{y}\to 0\;\tilde{y}\to \infty }\tilde{y}{\tilde{u}}^{\prime }={\left(cC\right)}^{-1},$$

which we recognize as the log law with *κ*=*c* *C* (cf. equation (2.5)).

To gain insight into the physical import of condition (5.3), it might be useful to turn to some of its implications aside from the classic laws. One such implication of (5.3), namely $0\leq F(\hat{y},\tilde{y})\leq F_{w}(\tilde{y})\leq C$ and $0\leq F(\hat{y},\tilde{y})\leq F_{d}(\hat{y})\leq C$, can be phrased as ‘the effect of finite domain and the effect of viscosity is to *slow down* the eddies that dominate turbulent shear-stress production in the flow’ and does no violence to physical intuition. Another implication of (5.3), $0\leq \tilde{y}F(\hat{y},\tilde{y})\leq \tilde{y}F_{w}(\tilde{y})\leq \tilde{y}C$ and $0\leq \tilde{y}F(\hat{y},\tilde{y})\leq \tilde{y}F_{d}(\hat{y})\leq \tilde{y}C$, or ‘the effect of finite domain and the effect of viscosity is to lessen the turbulent viscosity of the flow,’ may have a stronger purchase on intuition. (Note that $\tilde{y}F(\hat{y},\tilde{y})$ equals the turbulent viscosity of the flow normalized by *ν*, the kinematic viscosity of the fluid, as can be seen from (5.8).)

**Figure 2.**
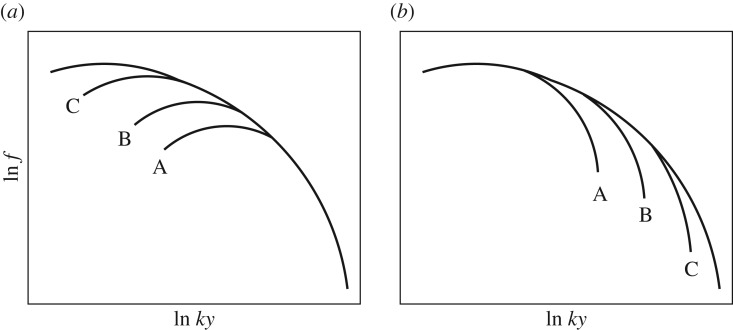
Typical plots of experimental and computational data on the dimensionless spectrum $f(ky,\hat{y},\tilde{y})$, drawn schematically after the original plots in [6], are used here to put condition (5.3) to the empirical test. (*a*) Plots of $f(ky,\hat{y},\tilde{y})$ versus *ky* for a fixed value of $\tilde{y}$ (the same for every plot) and four values of $\hat{y}$ (one for each plot). The plots collapse onto a master curve at high *ky*, in accord with the spectral analogue of the law of the wall (the master curve corresponds to $f_{w}(ky,\tilde{y})$). As *ky* is lessened, the plots peel off from the master curve in order of decreasing value of $\hat{y}$ (plot A followed by plot B, etc.); further, every plot remains below the master curve after peel off ($f(ky,\hat{y},\tilde{y})\leq f_{w}(ky,\tilde{y})$), consistent with (5.3). (*b*) Plots of $f(ky,\hat{y},\tilde{y})$ versus *ky* for a fixed value of $\hat{y}$ (the same for every plot) and four values of $\tilde{y}$ (one for each plot). The plots collapse onto a master curve at low *ky*, in accord with the spectral analogue of the defect law (the master curve corresponds to $f_{d}(ky,\hat{y})$). As *ky* is increased, the plots peel off from the master curve in order of increasing value of $\tilde{y}$ (plot A followed by plot B, etc.); further, every plot remains below the master curve after peel off ($f(ky,\hat{y},\tilde{y})\leq f_{d}(ky,\hat{y})$), consistent with (5.3).

But quite apart from such considerations, condition (5.3) can be put to the empirical test. In this regard, note that condition (5.3) presupposes the spectral analogues (3.2)–(3.4), which have been validated empirically by inspecting suitable plots of experimental and computational data on the spectrum [6]. Those very same plots, the essential features of which are reproduced in figure 2, can be used to validate condition (5.3), as we explain in the caption of that figure.

## 6. Discussion

To summarize, the classic laws can be predicated on (5.3), a sufficient condition concerning the manner in which $f(ky,\hat{y},\tilde{y})$ (the dimensionless spectrum of turbulent energy at a distance *y* from the wall) is affected by *δ* (the size of the turbulent domain) and *ν* (the viscosity of the fluid) via the dimensionless variables $\hat{y}$ and $\tilde{y}$, respectively, where $\hat{y}\equiv y/\delta$ and $\tilde{y}\equiv yu_{\tau }/\nu$. Broad in scope and agreeable to intuition, condition (5.3) is consistent with empirical data on the spectrum and may be deemed a general property of the energetics of wall turbulence.

We have shown that the classic laws of wall-bounded turbulent flows can be derived by relating the MVPs (which are the subject of the classic laws) to the spectrum of turbulent energy (which represents the distribution of turbulent energy among eddies of different sizes in a flow) without invoking any specific model of the spectrum. Our derivation has allowed us to conclude that the classic laws must be satisfied if a turbulent eddy cannot be energized by virtue of viscosity or finite domain, a condition that may be readily verified by empirical data on the spectrum of turbulent energy. Thus, we have been able to draw support for the classic laws from empirical data unrelated to the MVPs. From a broader perspective, our derivation indicates that, contrary to what might be inferred from the standard derivation of the classic laws, the MVPs as well as the attendant phenomenon of turbulent friction are inextricably linked to, and can indeed be interpreted as macroscopic manifestations of, the spectrum of turbulent energy [7,13–20].

## Data accessibility

This paper has no additional data.
